# Fair space? Community relations as a booster for improving resilience

**DOI:** 10.3389/fpubh.2025.1585985

**Published:** 2025-07-08

**Authors:** Coline van Everdingen, Peter Bob Peerenboom, Irene van de Giessen, Koos van der Velden, Philippe A. E. G. Delespaul

**Affiliations:** ^1^Department of Psychiatry and Neuropsychology, Faculty of Health, Medicine and Life Sciences, Mental Health and Neuroscience Research Institute (MHeNs), Maastricht University, Maastricht, Netherlands; ^2^Van Everdingen Health Care Research, Sittard, Netherlands; ^3^Tangram Health Care Consultancy, Doetinchem, Netherlands; ^4^RecoveryTalent, Middelburg, Netherlands; ^5^Department of Primary and Community Care, Radboud University Medical Centre Nijmegen, Nijmegen, Netherlands; ^6^Mondriaan Mental Health Centre, Heerlen, Netherlands

**Keywords:** user research, rights-based ecosystem approach, transdiagnostic assessments, dynamic monitoring, mutual learning, fair space

## Abstract

**Background:**

The opportunities of health and happiness are unequally distributed. Multiple triggers lead to social exclusion and can result in homelessness. Efforts are still failing to counter health inequity. Our ethnographic research explores the social pathways of citizens deprived of a home. How can we sustainably promote fair opportunities for all?

**Methods:**

Urgent information needs prompted field research among Dutch homeless service users. In semi-structured interviews, we collected information on health and needs. A transdiagnostic, generic strategy is used. Our focus is on the three themes of recovery (meaning, symptoms, and the social dimension), and their evolution over time. From a human rights perspective, the study examines the interactions between homeless service users and their environments. Case descriptions are added for a better understanding of dynamics.

**Results:**

The dual snowball sampling resulted in a representative sample of Dutch homeless service users in 2015–2017 (16 facilities, 436 users). The health and needs patterns reveal many interrelations of multiple needs. The user–system interactions uncover a systemic failure to match vital needs of the neediest. The interactions over time expose how social decline occurs and identify opportunities for improvement. The survival strategies show that stress often generates communication barriers, conflict, alienation and neglect. This has a negative impact on the self-image, (in)formal networks and place of users in society. A “deadlocked case” from a local experiment demonstrates how community relations can overcome modern system obstacles, while creating stable conditions for growth.

**Discussion:**

Despite abundant care and welfare facilities, many shelter users feel abandoned. Our rights-based ecosystem approach leads to the root causes of unfairness, inherent in the healthcare system and culture. The findings disclose that the resilience of Dutch society suffers (but can withstand) significant erosion. By extension, this applies to modern care systems in high-income countries. Fostering resilience requires meaningful social relationships that offer creative solutions. Exploring dynamics stimulates shared learning in safe and public spaces. Providing networks with concrete tools, the approach can foster a coherent, systematic commitment to enrich fair opportunities and pathways toward thriving.

## 1 Introduction

To date, opportunities of health and happiness are unequally distributed. Huge differences within and between countries exist (1, 2). Despite abundant knowledge and the best intentions, we fail to counter health inequity. So, what matters to enable improvement?

This synthesis presents the results from field research among Dutch citizens deprived of a home. The ethnographic research explores how environmental conditions, health and needs patterns, and dynamics over social pathways connect. From our background and perspective in public health and social psychiatry, we visualize important community relations and processes in an ecosystem approach (3–5). Ecosystem strategies consider people and their environments together. They monitor dynamics in various processes over time, while integrating information from multiple dimensions and perspectives. They also explore how people learn and develop resilience, building on universal “homeostatic rules.”

In daily life, families and other networks offer social environments for developing and mastering health and life skills. By nature, communities aim to preserve autonomy while supporting disadvantaged fellows. Transgenerational patterns and culture also affect growth dynamics over time. Ideally, governments secure essential conditions. International human rights treaties can provide guidelines (6). Besides, we have individual and shared knowledge how diverse conditions affect dynamics. This knowledge informs our understanding of how human capacities, autonomy, and meaningful relations develop (7–9). Environmental conditions matter: physical and social factors affect human resilience in communities and societies. Health disparities still lead to inequity. Social epidemiology has revealed the links between mental health issues, poverty, stigma, discrimination, and social exclusion (1, 10). Unsurprisingly, we have abundant evidence on effective health interventions and prevention strategies.

However, promoting health and growth remains challenging. Flexible resources (money, knowledge, prestige, power, and beneficial social protectors) have a significant impact on the social stratifications of population health and mortality rates (11). These “fundamental causes” gave reason for designing evidence-based care strategies and rights-based quality frameworks. The Convention on the Rights of People with Disabilities (2006), recognizes that disabilities are a social construct (6). While impairments are real, “disability results from the interaction between people with impairments and attitudinal and environmental barriers.” The WHO Commission on the Social Determinants of Health has addressed the unfairness of preventable health disadvantages. In 2008, she revealed how countries could promote health equity through comprehensive action on the fundamental causes of social determinants (1, 2)^.^ By visualizing the social stratifications of health, she demonstrated the inadequacy of economic growth and single-sector improvement. Increasing empowerment is essential to enable all citizens to live flourishing lives.

To date, significant disparities in fundamental growth-enabling conditions remain a pressing issue. Health inequity is everywhere (12, 13). Over the past decades, key policies furthered individualization, privatization, and austerity within market-driven healthcare systems (14). In parallel, solidarity began to shrink. Assuming universal freedom of choice, dominant narratives hold individuals responsible for their behavior and living situation. Illness is considered a manifestation of weakness, exposing people's failure to maintain autonomy. Despite extensive knowledge and good intentions, communities and countries massively fail to create a fair environment for growth. Disadvantaged groups mainly consist of people with complex needs (1, 15). Ultimately, social exclusion processes can lead into homelessness.

Homelessness is an extreme result of insufficient access to flexible resources. It reflects the interactions between citizens, institutions, and authorities in the cultural and macro-economic context of societies. Literature reveals that it results from a complex interplay between individual vulnerabilities, interpersonal, structural, and systemic environmental conditions (16–18). Various interrelated risk factors can trigger multiple pathways into social exclusion. For several reasons, the homelessness literature is fragmented and incomplete (19, 20). First, it is often domain specific and relying on administrative data. “Hard-to-reach” populations are underrepresented in social surveys. Due to the biomedical dominance and categorical health strategies, relevant health aspects are disregarded. Concurrent risks and structural factors are accentuated, while paying little attention to personal strengths or the human need of freedom. Vast evidence highlights the urgent need of integrated care programs for populations with multiple complex needs, such as homeless citizens with mental health problems (21–23). Studies of pathways toward thriving mainly reveal barriers and lacking preconditions for growth (24–28). Though ethnographic research increased the understanding of social exclusion pathways, few empirical studies have focused on opportunities to reverse the social decline (5, 29).

How can we bridge this gap? In theory, we know what can reduce health disparities or end homelessness. In answer to the “what-question,” the components are clear. This study centers the “how-question” through three objectives:

1. To describe the health of the Dutch homeless service users, including different health aspects and comorbidity;
2. To analyze patterns of marginalization and disclose care gaps in interactions of service users and systems; and
3. To identify conditions that promote recovery in marginalized populations with interdependent needs.

This synthesis outlines the socio-political context at the start of the study, in 2015. We describe the methods and the main findings. We discuss the current meaning of the research to its cultural-historical background. What matters to enrich fair opportunities and foster social pathways toward thriving?

## 2 A comprehensive ecosystem strategy

### 2.1 Urgent information needs require local health reviews

In 2014, a former Minister of Health was killed by a man in a psychotic state. The murder increased the public awareness of “confused people on the streets.” It pushed the debate about care quality and the roles of institutions in society (19). Instigated by the public debate, a national policy for restoring mental health related safety emerged. Meanwhile, the planned reforms continued, accompanied by austerity measures. The municipal responsibilities for disadvantaged and disabled groups increased. The socio-political call for action required local networks to bring the health and needs information on homeless service users up-to-date. After all, citizens without postal address are “invisible” in monitoring systems. Besides, systematic health assessments are not included in the basic routines of homeless services.

For service planning goals, homeless services and municipalities started to commission local health reviews. The largest service provider, The Salvation Army, was the first. As an independent researcher, one of us (CE) conducted the first review in the night shelters of two cities. The semi-structured interviews provided accurate insight in the users' health and needs, gave voice to their perceptions, and made the structural underservicedness transparent. The report fostered the regional and national debate. Since then, different local service networks ordered their own reviews. Successive reviews in a similar, participatory approach resulted in the HOmeless People Treatment and Recovery (HOP-TR) study.

Recruitment followed a dual snowball sampling (settings and subjects). Field research collected data in 16 facilities in seven cities, resulting in 436 interviews (19). The data collection was continued until saturation was reached. Comparing the background characteristics of our sample with Dutch reference data shows that it is a representative sample of the homeless service users in 2015–2017. The sample profile discloses that most were low educated (82.3%) males (81.0%) with a migration background (52.1%). Three out of four were roofless (sleeping rough or in night shelters). Most were previously homeless (78.8%); most were long-term or intermittently homeless at the time of the interview.

### 2.2 Comparing integrated assessments over various settings

Active in public health as a medical doctor, the interviewer (CE) was trained to conduct health and needs assessments. Equipped with open questions and several questionnaires, she participated in shelter life and merged with the service users. The narrative interview examined what happened over their lives and what mattered for future perspectives. The structured assessment included the interRAI Community Mental Health questionnaire. Worldwide, it was the first time this questionnaire was used in homeless settings. The *conjoint paper* describes the background, aims, and composition of the interRAI mental health questionnaires (30). Designed to support care planning in various settings, the assessments cover all life domains. Composite signal indicators allow a quick appraisal of well being, behavior, and daily functioning. The HOP-TR data is included in three histograms, that showcase possible applications. Comparing the Dutch homeless service users with homeless Canadian outpatients reveals similar patterns in the lifetime burden of adverse events. It shows comparable triggering rates of the self-care and harm-to-others indicators, higher than in the Canadian non-homeless groups.

### 2.3 What happens between homeless service users and community relations?

Next, the *methods paper* outlines the context and reasons why local service networks required their own reviews (19). Though Dutch care-welfare policy appeals to own capabilities of citizens in their networks, it intends to offer matching care for needs exceeding citizens' own resilience and resources. Therefore, the life histories and the living conditions of the users amazed us. Aware of the care gaps, many expressed their indignation or disappointment about the way they were treated. In their perspectives, services and/or society had let them down. The Community Mental Health questionnaire contributed to structured, systematical health, and needs assessments. What else could make relevant conditions transparent in the interactions between users and their environments?

The paper describes the comprehensive, rights-based ecosystem approach of the HOP-TR study. The field research collected user narratives in real life settings. It explores from various perspectives what happens between users and community relations. It uses a generic, transdiagnostic approach to health and needs. Collected in a life course perspective, the data covers the six dimensions of positive health (31).

**Figure 1:**
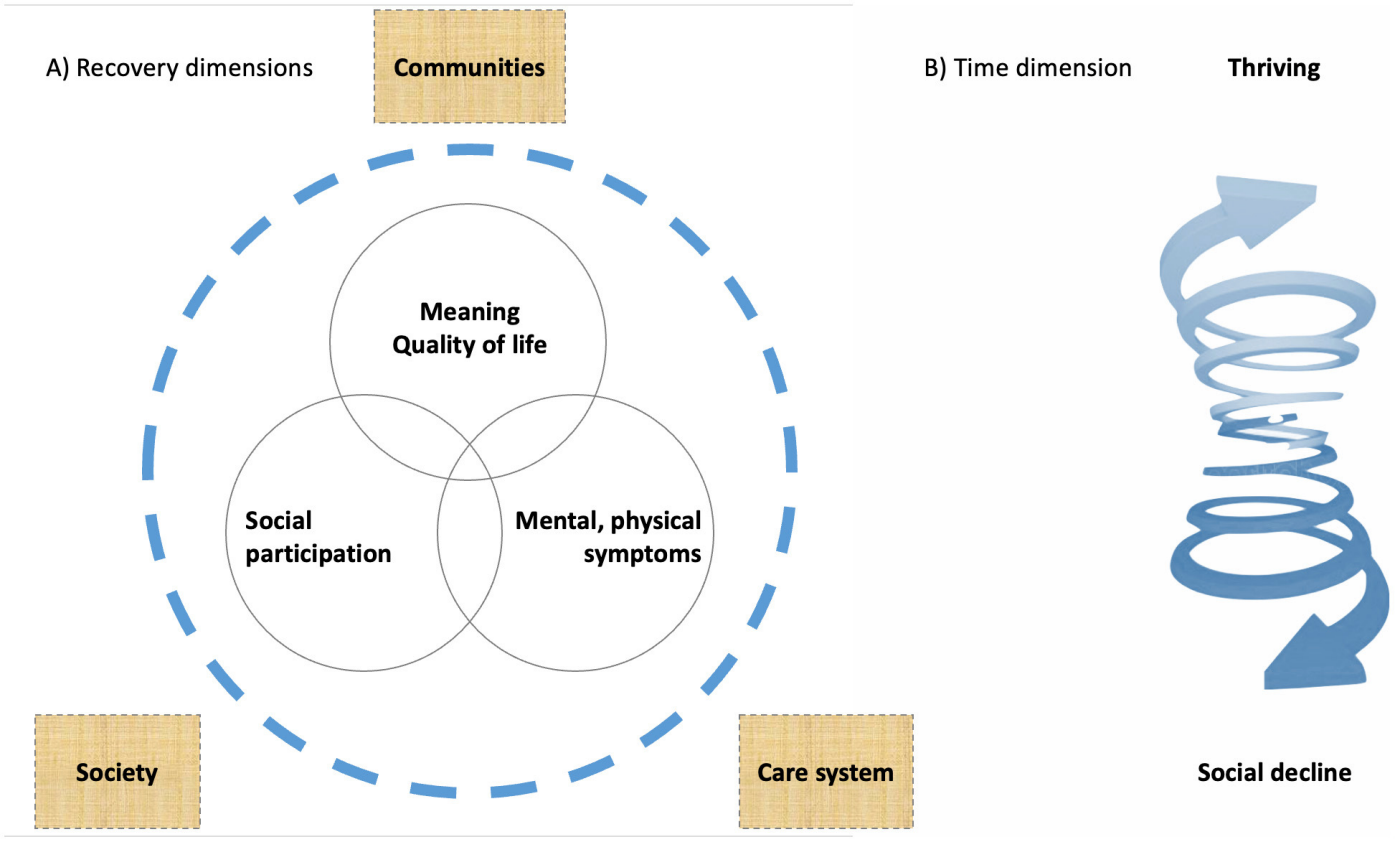
The three dimensions of recovery in relation to environmental conditions **(A)** and the dynamics in adaptional functioning over time **(B)**.

The interview data was structured in the (mental, physical) symptom, social (daily functioning, and participation), and personal (quality of life, meaning) dimensions of recovery (Figure 1a). This assists to explore how environmental conditions, health and needs patterns, and diverse processes over social pathways connect. We analyzed the life journeys through a “CHIME”-lens (Connectedness, Hope and optimism, Identity, Meaning, Empowerment) (32). The spiral (Figure 1b) represents dynamics. It reminds that multiple conditions and processes continually interact, while affecting the growth continuum between social decline and thriving. “Growth” evaluates (learning, development, and recovery) processes from a positive orientation toward thriving.

Initial analyses focus on the care/needs assessments of individual users. Additional analyses shift the focus from individuals to care networks and community relations. Combined analyses render a “country-case of citizens deprived of a home”. The essentials display how we gradually broaden the research focus (Supplementary Table S1). A casestudy of a local experiment is included, for a better understanding of dynamics. Seven vignettes, reflecting the life and worries of users like “Amin” and “Amanda,” illustrate the main findings (see Supplementary material).

## 3 A country-case of citizens deprived of a home

### 3.1 Health patterns reveal interdependent needs

Most service users (79%) had not visited any physician in the last 3 months. “*Health patterns*” presents the health symptom results (33). It depicts the physical and mental health perceptions, adversities, substance use, behavior, and social functioning in detail.

We examine the patterns of real symptoms that impact daily lives. The transdiagnostic mental health features reflect major complaints and behaviors, which describe current mental health over the past 3 months. The prevalences identify meaningful conditions relevant to daily functioning and to the course of participants' lives. In contrast to the categorical DSM-5 group diagnoses, implicit symptoms are not discarded. Similarly, we apply a pragmatic pattern strategy to examine the mental health-related care needs. A decision tree, based on the Dutch consensus SMI/EPA,^1^ supports the assessments. The consensus considers the impact of mental suffering in relation to natural homeostatic mechanisms and (personal and community) resources to overcome it. Typical of the natural course of SMI/EPA are the profound impact on daily life and the circular interrelations between symptoms in various life domains. Due to the circularity, the symptoms mutually trigger each other. The clusters of interdependent problems are difficult to overcome. People have integrated care needs if (1) pervasive mental health problems impact their personal functioning and participation in society; (2) interrelated symptoms and disabilities trigger each other; and (3) working relations that (assist to) integrate, and coordinate care are essential for fostering growth.

The results represent the character, extent, and overlap of health symptoms. Almost all users experience mental health problems (98.6%). The most frequent are addiction (78.0%), anxiety (75.2%), trauma (69.2%), and depression (67.0%). Intellectual (39.9%) and cognitive impairments (28.4%) are common. Most users (59.9%) have physical health problems.

The burden of health problems is high. A significant share (83.7%) must deal with four or more mental health problems (mean 5.3, SD 1.9, and range 0–10). The physical health burden is lower (mean 1.1, SD 1.2, and range 0–7), due to its focus on chronic conditions attended with monitoring needs. Nearly all (94.5%) have multiple health problems in two or more health domains (addiction, intellectual impairments, other mental, and physical health problems). So, the disease is extensive. Yet, present mental health problems not always require professional care. In this country-case, few users (5.3%) have no needs, some (22.3%) have temporary, conditional needs, and most (72.5%) have long-term, intensive needs attendant on severe mental illness. The prevalence of intensive, integrated care needs is high (72.5%) in comparison to the general population (1.7%). The circularity of the health patterns reveals the interdependent character of the needs. The narratives of “Paul” and “Paula” glimpse at the impact of intensive needs (see Supplementary material).

### 3.2 Local service responses to vital needs?

Local services aim to support disadvantaged groups in need of care. Still, many users were dissatisfied how they were treated. In the “*Vital needs*” paper we unpack the responses of local services to vital needs in Maslow's hierarchy (34). It describes how formal caregiver relations relate to present basic (administration, paid work) and (mental, physical) health needs.

First, all homeless service users need a home, mostly an independent place to live (92.7%). All have a history of financial problems; most (65.8%) report trade-offs regarding essential needs in the last month. Despite their need of paid work as a meaningful activity, most (55.6%) are persistently unemployed. Few (2.8%) have a regular job. The frequent paid work needs (82.3%) reflect the lack of certifications to sustainably acquire and hold a regular job. The administration needs (56.7%) uncover the prevalence of functional illiteracy. Besides, the daily impact of prior traumas, self-harm, and violence expressions is substantially higher among users with intensive care needs.

Nearly all users have conditional or intensive care needs because of their mental health (94.7%). Only 7.4% of the present mental care needs is matched in accordance with the consensus SMI/EPA. Most have both mental and physical health needs (69.3%).^2^ Only 2.3% with concurrent health needs receive appropriate mental and general healthcare. A marked share has concurrent basic needs (54.8%). Only 3.3% with concurrent basic needs receive adequate (administration and paid work) support. On top of the need of an income and a home, nearly all (92.9%) have vital health and/or basic needs in two, three, or four domains. Considering the various needs pairs in two domains, coverage by care is low (between 0.6 and 13.1%). Concurrent triple and quadruple needs are hardly met. Due to the multidomain character of the vital (health and/or basic) needs, the availability of matching care is extremely low. The underservicedness reveals the systemic failure of services to meet integrated care needs over various care domains.

### 3.3 Shaping conditions for recovery: an ethnographic policy evaluation in a city

The Dutch health-care system offers a broad array of services and interventions targeted at disadvantaged groups. All kinds of preventive and support programs are available in school, work, care, and community settings. What complicates the shaping of suitable arrangements for matching care needs over various domains? “*Shaping conditions*” reports on a local experiment to solve the care gap at the intersection of severe mental illness and homelessness (35). The public health context was enriched with a Special Assertive Outreach Team (“S-team”), targeted at citizens who continuously fall out of care. For several years, CE followed the team as a participant-observer.

The narrative data represents 15 referrals to the S-team. All were “deadlocked cases” with inadequate, stagnating care. Due to lacking opportunities, they were continually on the hotlist, but nothing happened anymore. In all cases, frustration and hopelessness in caregiver networks contributed to the referral. Their narratives unfold why interactions in highly specialized care arrangements get stuck. They show the harmful impact of unequal power and malleability assumptions to the reciprocal engagement in interpersonal relations and the human need for autonomy.

Moreover, the cases uncover what works for developing working relations. The S-team engaged in a similar approach with the cases and varying networks. Normal behavior, lived experience, and personal qualities (how to support growth/recovery processes) are the keys to establish collaborative relations in various settings. It stimulates listening to various perspectives, thinking in terms of solutions instead of impossibilities. Besides, the S-team uses socio-ecological system knowledge and metaphors for seeking alternative pathways. The involvement of the S-team marks new dynamics in mutual learning of diverse ecosystems.

All cases showed a similar, favorable course. Therefore, the narrative of “Simon,” a man living with psychoses, provides an in-depth demonstration of the results (see Supplementary material). As a child of regular substance user parents, he belonged to less advantaged groups in society. As a young adult, he landed on the streets and started to use. A decade with long hospitalizations followed. The power of professionals and their focus on restoring control defined his life. Mutual distrust induced alienation. He perceives hospital care as degrading and dehumanizing. His outcry conveys his lack of freedom, pointing to unequal power in treatment programs. He was over thirty, when he first moved to a place to live on his own. Since the S-team's involvement, it became clear that the admissions and seclusions had hampered developing normal prosocial relations and basic everyday skills.

The narrative revisits Simon's story in three sections (case history, care needs conceptions, and growth/recovery dynamics). The layered presentation shows how environmental conditions affect dynamics at inner-personal, interpersonal, community, and society levels. Despite his working relation with the S-team, parallel care plans from separate siloes appeared inadequate for ending the downward spiraling. The case proves the success of a triadic approach, that explores different perspectives and uncovers power imbalances in careseeker–caregiver interactions. Since then, Simon's collaboratives with important community relations are capable to diversify care. They sustainably preserve vital (basic and safety) conditions for emergent growth. Then, his work, hobbies, and meaningful relations give space for developing self-esteem and an adult identity.

### 3.4 Fair space for life: a dynamic monitor to facilitate growth

Returning to the country-case, “*Fair space for life*” describes users' survival strategies to the multiple stressors which make up their lives (36). Focused on human responses to stigmatization, discrimination, and other interpersonal rejections, the multi-motive model distinguishes prosocial, avoidant, and aggressive modes (37). Under low distress, the prosocial mode is prominent (PRM: 32.8%). High and protracted distress is found with an avoidant (AVM: 42.0%) or aggressive mode (AGM: 24.8%).

First, we compare background, mental health, and needs patterns by survival mode. With PRM, the mental health burden and daily life impact of previous traumas are lower compared to AVM-AGM. With AGM, nearly all are addicted and agitated. Intellectual impairments are also frequent. With AVM, depression, anxiety, and addiction are prominent. Half the PRM-copers has intensive care needs, compared to the majority with AVM-AGM. The coping-groups have similar physical monitoring, paid work, and administration needs.

Second, the responses disclose the intense impact of the continuing distress on the users' well being and behavior. The disease concerns are stronger with AVM-AGM. The substance use consistently increases from PRM to AVM and AGM. The disease raises internalizing and/or externalizing responses, such as self-harm thoughts, violent threatening, and violent acts. For instance, with AVM-AGM, one out of four report self-harm thoughts over the last year. With AGM, three out of four report physical violence over the last year.

Third, we visualize how distress affects important relations. The distress-induced dynamics over social pathways cause withdrawal, alienation, and social decline. Misunderstandings and conflicts end in rejections and stigma. At the inner-personal level, the distress badly affects self-confidence and meaning. Even with PRM, one out of three has no one they implicitly trust. At the interpersonal level, distress-induced dynamics generate frictions and conflicts. Externalizing distress increasingly impacts (in)formal caregiver relations. Likewise, rising internalizing distress particularly burdens informal caregivers. Correspondingly, four out of 10 with AVM have no informal helper at all. Further, the hospitalizations and police-justice contacts unfold what happens in interactions at community/society level. As expected, users with AVM-AGM are more experienced with psychiatric admissions than colleagues with PRM. Involuntary admissions more often occur with AGM. Chris' and Simon's explanations make their aggressive coping understandable (see Supplementary material). Striking is that all police-justice interactions significantly correlate with the rising externalizing distress expressions. Instead, no correlations with internalizing distress were found.

Fourth, we review vital conditions for health and citizenship. The systemic failure of the health-care system to deliver multi-domain integrated care puts the users at a disadvantage. In a life course perspective, the accumulating disadvantages increase. The life histories disclosed multiple reasons for disturbed school careers and unstable lives. Accordingly, the education level and work participation in all groups is extremely low. Over time, the AGM-group is most deprived. So, the results reveal that fair conditions are challenged by the cumulative impact of distress over the users' lives. The diverse sources of social exclusion are intrinsic to the health-care system and culture. For that reason, countering health inequity will require profound culture change.

## 4 Exploring alternative pathways for improving resilience

The persistent failure to reduce health inequity raises the question what matters for moving sustainably toward fair opportunities for all. This study examines the social pathways of Dutch homeless service users. Resilience is central. Starting from regular care practice, it records users' health and needs patterns through a generic, transdiagnostic approach. Next, it analyzes how user–system interactions affect growth dynamics on the continuum between social decline and thriving. Finally, it explores how social interactions can improve resilience.

Supplementary Table S1 outlines the essentials of our rights-based ecosystem filter. Transferring the focus from individual care pathways to community resilience, it examines how shared learning can enrich opportunities and pathways toward thriving. Previous sections document the methods and main study results. Here we contextualize the research to its cultural-historical background. The narrative analysis unpacks its contribution to modern understanding of resilience of individuals, communities, and society.

### 4.1 Health patterns reveal multiple interdependent needs

Building on shared knowledge, community resilience considers the resilience of (individuals within) communities. Since ancient times, city-states exclude “insane” citizens if necessary. For long, mental illnesses were considered well-defined entities with a fixed prognosis. In 1911, the humanistic psychiatrist Eugen Bleuler introduced a bio-psycho-social approach of mental health symptoms on a dynamic continuum between “normal” and “ill” (38). Despite its acceptance, it remained common use to “secure” mentally ill and disabled citizens in psychiatric asylums (39). In many countries, the dismantling of psychiatric hospitals fostered the public debate how to preserve community resilience. Starting in the American 50s, the community mental health movement marks changing interpretations of care roles by governments and citizens. In the eyes of co-citizens, policy makers, and institutions, the return at the scene of displaced persons demands alternative pathways of related communities to preserve safe living environments. Simultaneously, the release voiced the people concerned. Empowered by the recovery movement, it enabled them to express their perspectives and stand up for fairness. So, the dismantling stimulated new dynamics in the interactions between returning citizens, neighbors, and formal caregivers. Major recovery studies encourage to revise dominant visions on the role of medical treatment in growth processes. The American “Vermont study” is still a cornerstone (40). The three-decade study followed “100 revolving door clients” living with psychosis after their release. Published in 1987, the long-term results demonstrate how most of the “totally disabled, deadlocked cases” recovered and had meaningful community relations and activities in society. In parallel, Ciompi's three-decade research revealed the favorable impact of environmental conditions on course of mental illnesses in Europe (41). So, long-term research discloses how social pathways can offer alternative or additional enablers of emergent growth toward thriving.

Accordingly, homeless citizens with severe mental illness were targeted in the development of integrated community mental health models (21–23). Housing First with intensive or intermediate case management proved cross-culturally effective (42, 43). Shared decision making is central. Negotiating reasonable opportunities, the model supports consumer choice, self-determination, and community integration. Rent supplements and ambulant mental health teams are included, allowing to end homelessness rapidly.

Accordingly, the changes also affected the relations of various knowledge sources and disciplines, such as clinical, public, and community health. Since the 60s, psychological experiments of human stress responses increased the understanding of social learning processes. In 1994, Marmot substantiated that health differences within and between countries go beyond socio-economic differences, since social factors affect people's disease susceptibility (44). One year later, Link and Phelan's “fundamental cause theory” addressed that multiple interrelations between socioeconomic status, social support, and flexible resources affect and maintain the risks of disease (11). Observing epidemiological successes to identify plenty risk factors, they urged to contextualize individual risk profiles in relation to environmental conditions for growth. “Without careful attention, we run the risk of imposing individually-based intervention strategies that are ineffective and of missing opportunities to adopt broad-based societal interventions that could produce substantial health benefits for our citizens.”

At that time, cluster analyses of the patterns of shelter use in Philadelphia and New York revealed that the chronic shelter users (10%) consumed nearly half of all shelter days (45). The research exposed the interrelations of concurrent (addiction, mental, and/or physical) health problems with social exclusion in society. While overlap was nearly absent with transitional homelessness, it was prevalent with episodical and chronic homelessness. Subsequently, the cluster analyses were repeated in Denmark over 1999–2009 (46). Compared to the USA, the homelessness figures in Denmark were low. Even transitional shelter use concentrated among service users with mental health problems and substance use. So, the differing health profiles confirm that sociocultural and structural environmental conditions affect social exclusion processes.

In the UK, field research in a six-city survey (*n* = 1,286) disclosed the huge overlap of homelessness, substance use, institutional care, and “street culture activities” (47). Accordingly, the meta-analysis by Aldridge et al. (12) unpacked the multimorbidity and mortality costs of accumulating social exclusion mechanisms in “inclusion health populations” (homeless citizens, substance users, former prisoners, and sex workers). The extreme health inequity by far exceeded the expectations based on socio-economic status differences alone.

In the Netherlands, an ambitious homelessness strategy was active in 2006–2013 (48). Through tailored individual support trajectories, hands-on prevention strategies, and collaborative municipal stakeholder networks, it aimed to improve the living conditions of citizens at risk. Simultaneously, it intended to eradicate nuisance giving streets homelessness. The monitoring focused on eviction prevention, housing stability, and nuisance reduction. The “Coda-G4” study followed a cohort homeless adults included in municipal care trajectories over 2.5 years (49). The symptom inventories disclosed prevalent physical symptoms and mental distress compared to the general population. Substance use (46.8%), anxiety (45.3%), depression (41.3%), and intellectual impairments (29.5%) were common.

The Coda-G4 study documented the comorbidities attendant on intellectual impairments (50). Further, Amsterdam street doctor figures reported a “tri-morbidity” prevalence of addiction, mental, and physical health problems in 31% (51). The Rotterdam register demonstrated that mental health and addiction steadily explained 42%−43% of all street doctor consultations over 2006–2017 (52). This is in line with the reasons for non-elective hospitalizations in a large American inpatient sample (53). In the homeless group, mental health diagnoses comprised 41.1% of the admission reasons, compared to 4.2% in the non-homeless group.

Returning to this country-case, the research is rooted in a Dutch monitoring tradition that contextualizes perceptions and behavior to environmental conditions over time (54, 55). Over three decades, it uses “Experience Sampling Methods” for digital momentary explorations of relations between health perceptions and environmental conditions that facilitate empowerment and growth. Considering their heterogeneous, episodic character, it acknowledges that “multiple health symptoms in individuals arise not as function of a latent construct, but as a function of symptoms impacting on each other” (20). Therefore, transdiagnostic mental health strategies were proposed for recording meaningful health nuances. Acknowledgment of the circularity of mental health symptoms resulted in the Dutch consensus of SMI/EPA (56) and a novel mental health recovery movement (57).

As reported, the dual snowball sampling strategy rendered a representative country-case of citizens deprived of a home. The comprehensive assessments yield a rich multidimensional data-set on the symptom, social, and meaning dimensions of health and growth over time. Though visionary papers proposed transdiagnostic mental health strategies, the practical use is low (123). As far as we know, this is the first country-case that represents mental health in a transdiagnostic approach. So, what insights come from the users' health and needs patterns?

First, the detailed data record the character, burden, and overlap of concurrent health symptoms that affect their lives. Where prior figures were based on categorical health classifications, the pragmatic, pattern-oriented assessments yield higher prevalences. The heterogeneous data portrays the inter-symptom relations and the temporal intra-symptom dynamics over time. It allows an integral appraisal of various health dimensions, while recording the real impact of concurrent health-related needs.

Second, the literature at the intersection of severe mental illness and homelessness accentuates the complexity of the needs (16–18). The health patterns reveal that “multiple complex needs” are often “multiple interdependent needs.” Complexity evolves from the circularity in the needs' clusters, as illustrated in the vignettes. Simultaneously, the differentiation between conditional and intensive needs visualizes the higher impact of prior traumas with intensive needs. The users' share with intensive needs is over 40 times higher than in the general population. Capturing the circularity, the label “intensive” accentuates that the needs demand patience and multiple chances from robust, relation-driven, and process-oriented care strategies.

Finally, the data reveals high prevalences of interdependent vital needs. The missing of paid work support in care programs (28) suggests that homeless citizens are incapable of fulfilling regular jobs? According to professional care standards, the responsiveness to vital needs reveals a pervasive neglect. What complicates the shaping of suitable arrangements, focusing on integrated care needs over various domains?

### 4.2 Distress-induced dynamics trigger social exclusion

Synthesizing relevant knowledge, John S. Strauss spent his entire life studying human interactions to better understand health and growth (40, 58). Educated by Jean Piaget, he understood that “the very nature of life is constantly to overtake itself.” Aged 85, he published a paper with two narratives of the life and death of his mother (59). The first one portrays the health symptoms in a biopsychosocial perspective. The second one enriches the factual events with subjective data about personal feelings, interpersonal relationships, and meaningful social activities in various networks. The changed perspective sheds a different light on the same case history. Strauss argues that the traditional (biopsychosocial) approach cannot reflect “… the power and nuance of experience, feeling and decisions.” The subjective perspective acknowledges individuals as the key persons of their own lives, including their perceptions and choices. He discusses metaphors and complex systems thinking as process tools for a better understanding of behavior, suffering, and emergent growth.

Similarly, moving psychiatric patients into the community, the dismantling of the hospitals enriched the public debate with the voices of “displaced persons” (39). Fighting against imposed treatments and coercive care, user/survivor voices substantiated that people should remain the owners of their own choices. Alongside the user/survivor movement and the “mad movement,” peer research emerged (60). For instance, the Canadian At Home Chez Soi study (2009–2013) used a broad, multidimensional approach to evaluate the diverse effects of Housing First with case management for citizens with homelessness and severe mental illness (61). Its “Peer Qualitative Research Group” aimed to go beyond consultation and collaboration. Voronka et al. documented how the peer researchers reclaimed their roles as knowledge producers by providing clarity about (un)helpful help. Their interviews disclosed that the need of a clinical diagnosis for receiving social support caused disconnections. Clinical, oppressive, and institutional help were unhelpful. Instead, throwing out the textbook and mutual aid was helpful. Later Voronka shares her perceptions “as a madwoman” in the co-creation of knowledge (62). Her auto-ethnography unfolds the differences between “the origins story”, hearer's perspectives, and the co-produced epistemes about resilient and recovered subjects. Through dominant power dynamics over relations, the metanarratives tend to reduce counter-discourses from user/survivor voices to inferior knowledge: “Working with/in dominant understandings of mental health/illness, I continue to invite co-citizens to think behind binaries. Such encounters seem like dialogues, but my capacity to be recognized beyond the psychiatric gaze is limited…” Llewellyn-Beardsley et al. (15) promote to include the voices of people experiencing structural inequalities in research. Alert to the loss of emancipatory power attendant on the ways narratives are used, they plead for performative analyses of storytelling in its immediate, socio-cultural, and historical contexts.

Aimed at enabling improvements, the six-city survey elicited the term “multiple exclusion homelessness.” It induced social-epidemiological research of the risk of homelessness (47). The regression models defined the contribution of individual (background, vulnerabilities, and behavior), social support, and structural (labor and housing market) factors to social exclusion pathways. Poverty factors explained half the variance in the UK.

Likewise, the risk of homelessness in Denmark (46) was mainly affected by concurrent psychosocial vulnerabilities (mental illness, drug abuse, alcohol abuse, and previous imprisonment). The contribution of income-related factors (low income, unemployment, and low education) was less. Benjaminsen concluded that “the otherwise strong safety net is apparently not tight enough for the most socially vulnerable groups.” The problematic, recurrently homeless groups nearly completely consisted of single male substance users.

Moreover, ethnographies in many countries demonstrated differing conceptions and power imbalances in the engagement between users and service networks (25–27). Still, social determinants and compromised systems cause instabilities in interpersonal and interorganizational interactions, funneling into downward spirals. Research confirmed the high prevalence of adverse childhood experiences and revealed insecure attachments in all participants of a group with multiple exclusion homelessness (63, 64). The “Power Threat Meaning Framework” provides a meta-framework for identifying patterns in emotional distress, unusual experiences, and troubling behavior (65). It equally integrates knowledge from various sciences, to overcome the medicalization of social and existential needs. Further, research identified practical barriers in care for homeless citizens with mental illness in 14 European capitals (24). Insurance problems, limited opening hours, insufficient outreach services and qualified mental health staff were common. Besides, lacking coordination and prejudice caused collaborative problems. Instead, mutual trust and an unintrusive attitude are essential.

The Dutch Coda-G4 study disclosed that individual support trajectories produce significant improvements in the resources for basic needs, access to social rights, social participation, and police-justice contacts (66). The independently living group perceived more autonomy and relatedness than the institutionalized group (67). Further, the group-based “growth-through-participation” intervention improved the social participation, self-esteem, distress, and quality of life (68). Goal-oriented working relations have a positive correlation with perceived belongingness, self-esteem, strengths, and informal support of homeless clients (69). The study concentrates on outreach workers, but normal interhuman contact with pragmatic support offers an alternative explanation of the positive effects.

In this study, many users shared that society, services, or important others have let them down (19). With the spiral in mind, we investigate what happens between users and community relations over time.

First, the vignettes show that common social challenges, disadvantages, and lacking resources push social exclusion. After his family had failed in offering him a safe place, Simon's narrative reveals that consecutive traumas during many admissions and seclusions continued (see Supplementary material). It unpacks how individualistic, professional-driven care overlooks environmental conditions for growth. Consequently, misunderstandings or other disturbances escalate care into highly-specialized care. This leads to mutual frustrations and alienation, while impoverishing his opportunities for growth. Repeatedly, Simon resisted the unfairness in enforced treatment. Despite growing local awareness of the successful approach, it demonstrates the ineffectiveness and costs of elaborated, overregulated systems. In the country-case, similar distress-induced dynamics cause interaction problems at various ecosystem levels. Unfair assumptions hold users responsible of their situation, disregarding the detrimental impact of environmental conditions and cumulative disadvantages over time. All coping groups are dealing with systemic underservicedness, so their survival strategies are “understandable” responses to pervasive neglect.

Second, the Dutch patterns of adversities and struggles with “troubling behavior” are similar to the UK. The main drivers toward social decline are identical to the UK and Denmark. The country-case portrays an extensive overregulated health-care system. After all, one would expect more interaction problems with AVM-AGM than with PRM. Emerging from the absence of substantial differences between coping groups in the “serviced-needs-if-needs-were-present”,^3^ the health-care system to some degree accommodates increasing needs. Besides, the serviced-needs by mental health-related care needs^4^ display that the presence of outreach services and community treatment teams responds to increasing needs levels. However, the overregulated systems hinder access to natural community resources. The vignettes illustrate the paradoxical, detrimental impact of care in a modern welfare state. Fred's history reminds that the need of a home can be resolved quickly (see Supplementary material). Moreover, the consistent correlations of all police-justice contacts with increasing externalizing distress expressions^5^ demonstrate the systematic focus on control. Consequently, the dynamics over social pathways lead into social decline. This empirical data shows that the users have good reasons for dissatisfaction. So, what can be learned from this country-case on opportunities that promote social pathways toward thriving?

### 4.3 Exploring dynamics and shared learning over social pathways

Due to recurrent crises in the past decade, the dynamics of multiple conditions and processes that enable resilience gain growing interest (5). In socio-ecological visions, homeostatic mechanisms continually record, integrate, and assimilate information from various sources and perspectives. So, our responses to internal and external environments continually adapt and adjust to preserve resilience and enable growth. Focusing on our needs for competence, relatedness and autonomy, the “self-determination theory” still contributes to modern understanding what matters (8). Ordering self-regulation styles and perceived locus of control by various motivation sources, its taxonomy helps to understand responses to imposed regulations or perceived power imbalances. Further, the tripartite model concentrates on the coherence, purpose, and significance in interactions for measuring meaning in life (70).

Social interactions build shared values, solidarity, and collective conscience (9, 71–73). People are more prone to change behavior when seeing liked and trusted others changing theirs. Shteynberg et al. (9) observe that individual learning (from others) complements collective learning (with others). Collective attention to information builds common knowledge and social cohesion. This founds mutual understanding what matters, while giving direction to collaborative actions. “Collective attention expands the chances of successful collaboration, as it leverages the power and knowledge of multiple minds to produce superior cultural innovations.” Thus, shared learning and mutual understanding open community resources and enrich opportunities toward thriving. Community groups can provide “safe spaces” that ease to share and reflect on crucial conditions for growth (74). Then critical thinking allows finetuning of homeostatic thermometers. Reflecting on dynamics in inner dialogs can help to internalize and deepen insights. Likewise, public spaces constitute an important link between all kinds of community groups, among others due to numerous spontaneous interactions with (un)known others (75).

Despite efforts, mental health problems elicit stigmatization, discrimination, and other interpersonal rejections. Therefore, modern care models implement resource groups (76–78). In such groups, meaningful (in)formal caregiver relations collaborate for arranging triadic responses to symptomatic, social, and meaning dimensions of present needs. Dignity and mutuality in contacts are essential (79). Recovery colleges and other user/survivor communities are dedicated to improving conditions for growth, both publicly and in safe spaces (80, 81). Still, Campbell warned that the “partnership mold” in collaboratives may mask underlying causes of unequal opportunities (74). Providing global examples, she explained how “advantageous social networks” found a more critical, power-focused understanding of social capital. In safe spaces, “bonding” and “bridging” within excluded groups promotes solidarity and fosters growth. Besides, “linking” to more powerful champions can boost “the pushes from below” beyond local communities. The examples depict how grassroot initiatives promote inner dialogs, community dialogs, and public debate, while affecting (sometimes resetting) opportunities for growth. Recently, the COVID-2019 pandemic demonstrated that *translocal learning* can assist resistance, emancipation, and transformation of social norms beyond communities, institutions, and geographical regions (82).

Aimed at enabling improvements, in 2008 the final report of the WHO Commission on the Social Determinants of Health conveyed a clear call: “closing the gap in a generation: health equity though action on the social determinants of health”(2). She warned that successful strategies go beyond the health-care system, as they require material support for a decent life, self-determination and control, and political will. Several years later, Brassolotto et al. (83) interrogated public health workers in Toronto how they applied the social determinants. They acknowledged that “past worldviews and thinking patterns can serve as obstacles to future progress and knowledge production.” Working on transformations appeals to conceptual understanding, but also touches moral norms, role conceptions, and power dynamics with concerned citizen groups. From their biomedical approach to risk factors, workers in “functional units” focused on individualized preventive actions. In their opinion, broader actions to the socio-political contexts of social determinants fell beyond their span of control. Conversely, workers in “structural units” engaged in collaborative actions in diverse settings, naturally contextualizing social determinants to relevant socio-cultural-historical backgrounds.

Epistemology is the philosophical study of the nature, origin, and limits of human knowledge.^6^ Aware of the potential impact of differing visions, Plamondon et al. (84) conducted a scoping review how the Commission's actionable recommendations were integrated in knowledge-to-action plans. Nearly half the papers over 2000–2016 refrained from contextualizing inequity results to socio-cultural-historical backgrounds. One third documented empirical research or program evaluations. Though the action-orientation after 2008 increased, epistemological obstacles related to individualist, biomedical, neoliberal ideologies narrowed the dominant discourse. Recognizing the dynamic interrelations between global, national, and local determinants, Krumeich and Meershoek (29) identified the major challenges to overcome structural social conditions and counter health inequity. Dijkstra and Horstman (85) demonstrated that dominant language and individualized visions in European policy reports contributed to the problematization and reification of lower socio-economic status groups. The meta-narratives attendant on the small stories of volunteers, welfare and municipal officers reveal the power dynamics in the construction of the Dutch “participation society” (86). The different stories disclose the democratic potential of citizens' representation and transformation of responses to community concerns (87). They employ the “social sewerage system” as a metaphor of the social dimension (88). As a commodity, it doesn't end in poor neighborhoods; it reconfirms that all citizens are equal.

With Link and Phelan's warning in mind, these epistemological challenges underpin the need of coherent strategies for shaping favorable conditions for growth (89–91). Within health-care systems, the “model for improvement” is widely used (92, 93). Building on data-driven consensual choices, its Plan-Do-Study-Act cycle promotes focused actions, joint evaluations, and readjustments over time. Due to its universal, iterative character, it is a powerful instrument for shared learning in teams or collaborative networks. Embedded in participatory research, the cycle can foster reflection-in-action, emancipation, and empowerment in communities as well (94–97).

Regarding citizen groups with multiple interdependent needs, it is undisputed that shared learning is a team sport (96–98). This requires partnerships at various ecosystem levels on an equal, integrative footing. The Vermont study is a successful example of participatory research for social change, due to consistent long-term investments in collaborative multi-stakeholder networks (99, 100). Later, spurred by the Vancouver Olympics, public worries about the North-American community health failure created an opportunity window for the pan-Canadian At Home Chez Soi “demonstration project” (101). Surrounded by action-, peer qualitative, and randomized controlled research, it is a successful country-example of a collaborative co-creation process. European efforts to create opportunity windows for implementing Housing First appeared less successful. Where the Canadian initiative was deeply rooted in action-research driven practices, the French project was constructed from a technocratic strategy (102). Although the Danish homelessness strategy represents one of Europe's largest Housing First Programs, only one out of twenty homeless citizens were served (103). Standing in a national tradition of collaborative community initiatives, the Housing First strategy in Finland seems more effective (104).

Equally, the Dutch homelessness strategy is technocratic. Focusing on public safety, all municipalities have service desks for alerting public order systems to confused people in the streets. Visible homelessness in Dutch cities is low, compared to many other western countries. Therefore, we explore what happens when users engage with care systems.

From professional caregivers' perspectives, our data reflects how local service networks must navigate through the maze of regulations, searching for effective ways to deliver “good care.” The histories portray how they continually struggle with dichotomous access criteria that complicate to reach and serve the neediest. After all, they are appointed to handle the system rules. For dozens of reasons, each care silo primarily focuses on its own specialistic care business (105). More robust expertise how to shape integrating plans over various care domains is inadequate and cannot develop (106). Municipalities should coordinate the connections between various health-care domains, but lack power and practical knowledge in specific relevant domains. Despite ratified universal health coverage, research shows system failure that threatens the right to health (107).

Likewise, from community perspectives, overregulated, siloed systems hamper access to natural resources. Burdening rules curtail problem-solving skills and resilience. Citizens expect to be serviced. People who dare to use fuzzy space for their own community solutions, risk being accused of fraud. People like Amin and Amanda, who do not fully understand the Dutch language and system rules, are more at risk to make mistakes or become victims of financial abuse (see Supplementary material). For instance, the handling of the childcare benefits act by the Tax Administration resulted in a political scandal. Meanwhile, the automated charging with social insurance contributions of people without an address simply continues. Unpaid fines and tax bills automatically produce increasing debts, without checking whether the original charge notifications had ever reached them. Amin's history illustrates the criminalizing results of well-intended mistakes. So, legitime appeals to social rights may result in punishments for fraud. Hindered by bureaucracy, debt counselors, services, and municipalities have great difficulties in finding simple, pragmatic solutions in such situations. Many users preferred seeking their own way, outside of the service systems, instead of accepting imposed system rules they have learned not to trust. In all, those interactions disclose how overregulated systems disempower workers and citizens in communities. Consequently, their resilience and alternative opportunities decrease.

A telephone survey in eight European countries disclosed a lifetime prevalence of homelessness of 5% (108). In the Netherlands, 4.5% had ever slept rough or in a homeless shelter. The lower prevalence (<2%) in the official estimates is explained by its focus on visible, nuisance giving homelessness (109).

To date, the Dutch homelessness research and action plans highlighted disadvantages and social exclusion (48, 110). This country-case demonstrates that the multiple interdependent needs are systematically accompanied by interaction problems with important community relations. It reveals how elaborated, overregulated systems complicate adequate responses to vital needs of the neediest. Instead, dominant assumptions create and maintain unfair conditions for growth. The dynamics over social pathways make the root causes of health inequity manifest, intrinsic to the health-care system and culture. For long, mistrust is deeply rooted in the Dutch society (111). The strong focus on control lacks a readiness to accept uncertainties and invalidates individuals to explore alternative pathways. This limits the acceptance of diversity. Besides, the distress-induced dynamics over social pathways disclose a limited understanding of fundamental conditions of emergent growth. The Dutch society still faces many epistemological obstacles, which largely connect to the dominant, individualized biomedical visions of health and growth.

Whereas, this study represents citizens deprived of a home, the dynamics in interactions uncover universal shared learning challenges for developing adequate responses to dynamic, multidimensional needs. After obtaining a new home, most strengths and needs remain unchanged. Inherent in the connections and circularity in needs clusters, non-homeless citizens with multiple interdependent needs experience similar interaction problems with systems. Ethnographic research explored the community mental health responses to psychiatric crises in Utrecht and Trieste (112, 113). In Utrecht, differing conceptions between workers with various backgrounds elicited frictions and misunderstandings. Seeking how to arrange good care, the Dutch team used an individual-functional, the Italian a community-ecosystem approach. In local settings, triadic collaborative networks proved effective to manage collaborative challenges and diversify care, while releasing all community resources (35, 77, 114–116). Recent policy reports support municipalities and social domain stakeholders to acknowledge the importance of reciprocity and ownership for strengthening the social infrastructure in neighborhoods (117). In practice, developing shared repertoires that successfully merge symptomatic with social objectives remains challenging.

Looking at the difficulties in interactions between systems and citizens deprived of a home, we can assume such challenges occur in a (much) larger group of citizens than the 0.02% officially homeless or the 1.7% citizens with severe mental illness (SMI/EPA). Observing the differing interpretations of social rights between citizens and institutions, many governmental bodies signaled common system fails for citizens with multiple multi-domain needs. Repeatedly, the Netherlands Court of Audit warned of the practical problems which complicate administrative processes for implementing the elaborated governmental rules. The National Ombudsman documented the illogical consequences, illustrating that the practical implementation further disadvantaged citizen groups (118).

After the COVID-19 pandemic, the State Council of Health and Society recognized that health inequalities increased and affected an increasing group (119, 120). Pleading for fundamental health-care transformations, she identified priorities for restoring the resilience of citizens and society. Commemorating the public health impact of closed sewerage systems in the 19th century, she accentuated the collective responsibility for living environments, that naturally offer a dignified existence and health for all. Starting from health-care needs of communities, she encouraged citizen-caregiver networks to co-create opportunities for integrating health-care responses to present needs. Simultaneously, she recognizes that such explorations demand radical changes in the governance, financing, and accountability mechanisms.

Our rights-based ecosystem filter makes essential conditions for health and citizenship transparent. The rights of a home and a standard of living are top priorities but remain structurally unmatched. This requires a profound culture change. Considering common struggles in modern socio-cultural contexts, we argue that citizens deprived of a home are prototypes of citizens with multiple interdependent needs in high-income countries. Naturally, serving (non)homeless populations with such needs is no routine care. The complex dynamics in community relations explain why the social-sewerage system is defect. Moreover, dynamics also reveal crucial opportunities for improvement.

First, our country-case reveals the power of normal behavior and socio-ecological system rules to engage and maintain sound relations with citizens with multiple interdependent needs. As shown, the vignettes portray users' perceptions and survival strategies to cope with daily challenges. Their voices show that meaningful community relations found hope for the future, even if single contacts are left.

Second, it subscribes the significance of mutual learning. It discloses that other community relations can function as “growth incubators” later in life, if the primary family has failed. Even in the most “deadlocked cases,” triadic collaboratives can effectively shape conditions for emergent growth. It demonstrates how process-oriented interactions in triadic collaboratives expand capabilities for dealing with vulnerabilities, while increasing community resilience. Listening to people in their daily (socio-cultural-historical) contexts, this data confirms the need of “advantageous social networks.” The mental health burden and the prevalent intensive care needs substantiate the importance of safe spaces and finetuned antennas for fostering mutual understanding of vital conditions for growth.

Above all, it demands coherent planning-and-control cycles at all ecosystem-levels, while focusing on fair space for life. Recruitment reflects that dynamic monitoring in community-based participatory research can facilitate shared learning, empowerment, and emancipation in diverse settings and systems. Assessments display how rights-based ecosystem monitoring can make power dynamics, tacit knowledge, and unnoticed resources in community relations transparent. The pervasive neglect urges to explore alternative strategies for developing fair space. It encourages to promote collective learning. This elicits dialogs what is essential and acceptable. The rights-based ecosystem filter returns the question how to preserve resilience to communities and society, including the people concerned. Making dynamics transparent, it stimulates mutual learning in diverse socio-cultural contexts what matters for resilience.

## 5 Methodological considerations

The HOP-TR study concentrates on a sample of Dutch homeless service users. Intermittent and long-term service users are overrepresented, because of the cross-sectional design. In Dutch population health surveys, citizens without a postal address are underrepresented. Stratification is based on home address or data is retrieved from administrative databases.

The Coda-G4 study documents care prevalences of homeless citizens included in municipal care trajectories. Conversely, our research presents population prevalences based on field research among homeless-service-using citizens, irrespective of their inclusion in care trajectories.

As mentioned, all research data was collected by a single interviewer. The data of the local experiment is cohort data collected over 2.5 years. The HOP-TR data contains only cross-sectional data of single interviews. So, the quality of the HOP-TR data is limited to face-to-face encounters and observations during the local reviews. Still, the ethnographic HOP-TR research yielded a rich narrative dataset. Recent health information was hardly available, but the interviewer's medical background enabled her to collect and interpret actual health and needs data. The research focuses on the health and needs patterns. As everything relates to everything, we only consider highly significant correlations (*p* < 0.01).

Naturally, the convenience of the sample requires a careful appraisal. The life histories show that homeless service using episodes often alternate with other periods of homelessness and (sheltered) living. So, the homeless service users of this country-case are part of a larger, diverse group with insecure or inadequate housing. Accordingly, recent point-in-time-counts include variants such as “sofa-surfers,” long-term “camping-campers,” and “work-migrants-lodging-in-holiday-parks” (121). In the latest counts, one out of three are women, nearly one out of five minors (122).

Besides, the time span since the start might raise the question if the data is up-to-date. Since 2015, the extent and urgency of the lack of affordable housing increased. For various reasons, it's likely that the composition of citizen groups faced with homelessness changed. On the other hand, no structural policy changes occurred. The overregulated systems remained, while the tension within systems even increased. Looking at the contextual evidence, there is no reason to assume that the dynamics over social pathways dissolved.

This paper is founded on a unique dataset, that gives a voice and a face to “invisible citizens” at the edge of society. Since the start, the interviewer (CE) continued listening to the people concerned in their daily environments. The longitudinal ethnography of the local experiment increased the hermeneutic consensual understanding of the HOP-TR data. Conducting the research without additional funding extended our opportunities to explore the meaning of this data optimally.

## 6 Conclusions

### 6.1 Mutual learning on opportunities for improving resilience

Considering the persistent failure to counter health inequity, this synthesis aims to contribute to modern understanding of resilience. Based on ethnographic research among Dutch homeless service users, we adopt a rights-based ecosystem filter. Transferring the focus from individual care pathways to community resilience, it explores how mutual learning can enrich opportunities toward thriving.

A dual snowball sampling strategy resulted in a representative offset of homeless services and service-using citizens deprived of a home. The generic, transdiagnostic assessments clearly depict the character, burden and overlap of present health problems. “Multiple complex needs” are “multiple interdependent needs”. The mental health-related care needs make the circularity transparent: most (72.5%) have intensive needs; 40 times more than in the general population. This is both the cause and effect of the limited growth opportunities.

Interactions depict what happens between users and systems. The rights-based assessments uncover the systemic failure of Dutch service networks to match vital multidomain needs. Though nearly all users have integrating needs in two or more domains, appropriate care is lacking. Over time, accumulating disadvantages increase. The dominant (individualized, biomedical) care approach creates exclusion criteria for comorbid conditions and disregards the role of environmental conditions for growth. In this process of failures, users are labeled as non-responders. The society reacts with technocratic control and containment actions, often leading to social decline, herewith hampering access to natural community resources. Consequently, distress-induced dynamics cause interaction problems at various ecosystem levels. Misunderstandings or other disturbances further escalate care into highly-specialized care.

Our study shows the costs of elaborated, overregulated systems. The power dynamics between community relations make the root causes of the disabilities transparent, intrinsic to the health-care system and culture. At the same time, our study reveals the meaning and opportunities from community relations. Normal interhuman behavior and socio-ecological system knowledge can help to develop working relations. Triadic networks, that link different domains and integrate scattered care resources, are crucial to manage collaborative challenges and diversify care. This empowers the use of available community resources.

Those findings demonstrate how mutual learning in collaborative networks can enrich shared knowledge and commitment to community conditions for growth. Our rights-based ecosystem filter redefines collaborative challenges between community partners. It reconnects with ancient knowledge on the power of triadic dialogical leadership on an equal integrative footing. Seeking how to improve environmental conditions, we can build on extensive knowledge of the social determinants of health. Proven effective interventions and prevention strategies direct actual improvement efforts for health and well being. Despite that, this “country-case” reveals a systemic failure to match vital multidomain needs of the neediest. The assessments in three (symptom, social, and meaning) dimensions already make some causes of health inequity manifest. Adding the time dimension leads to the root causes of the unfairness. Recognizing that the system obstacles and opportunities are everywhere, we conclude that the social resilience of Dutch society suffers, but can withstand significant erosion. By extension, this applies to modern care systems in high-income countries.

This study discloses how social pathways can offer alternative or additional means that facilitate growth processes toward thriving. It reveals that the insights of prior three-decade studies are still up-to-date. Those findings demand for a profound culture change. This requires a shift in our thinking: from individual care to community resilience. From a rights-based perspective, our ecosystem filter explores what enables change by approaching disabilities as a social construct. It provides communities and societies with necessary insights and process-tools how to create fair space and enrich opportunities for growth. Therefore, it digs into ways to facilitate mutual learning and foster empowerment. Its focus on the dynamics in community relations can facilitate shared learning between individuals, care resources and society at large. The social decline—thriving continuum stimulates communities to explore opportunities for growth. The narratives of citizens deprived of a home reveal generalizable knowledge of more collaborative strategies to collect and share information. Mutual learning in safe spaces and public spaces complement and boost each other. Therefore, the study expands modern understanding how we can pave pathways to thriving for all.

### 6.2 The modern challenges in seven transitions

Our rights-based ecosystem filter examines the resilience of citizens in communities. It enables to replace the fighting of health inequity with positive attention how we can enrich opportunities toward thriving. Merging ancient socio-ecological knowledge with recent psychological insights, the first version of Table 1 was drafted while we analyzed user–system interactions and assessed user's survival modes. It characterizes interaction patterns between users and care networks. It compares user–system interactions in an individualized biomedical with a rights-based ecosystem approach to care. The dimensions display various aspects of the information/knowledge exchange underlying to care performance, that allow mutual explorations and negotiations on one's role in what requires attention or action.

**Table 1:** Knowledge exchange between users and care networks.

**Aspects of knowledge exchange**	**Individualized biomedical approach**	**Rights-based ecosystem approach**
Action arena	*Interpersonal relations in formal care contexts* Individual education and disease management arrangements. Ideally, informal carers are involved	*Triadic community relations in daily life contexts* Providing fair space for life. Caring for each other from mutual relations: merging resources from individuals and (in)formal caregivers
Power and resource	*Professionals in institutions* Highly individualized care. Professional expertise is leading. The dominance of professional power generates power imbalances	*Citizens in communities* Mutual sharing of experiences in communities opens novel perspectives and pathways Natural, reciprocal interactions promote equity
Focus	*Biomedical precise diagnostics* Biomedical approach: considering individuals, to identify and fix the causes of growth issues, defining time-limited care needs to restore or improve health	*Transdiagnostic, contextual diagnostics* Strength-based socio-ecological approach: considering individuals in communities, alert to concurrent, interdependent needs, exploring environmental conditions that enable emergent growth
Standards and boundaries	*Dense, entangled specialistic norms* Evidence-based quality guidelines on separate problems Unfamiliar, distant human rights conventions	*Robust, rights-based norms* Normal, reciprocal behavior Respect for dignity and autonomy (equity) Process-oriented capabilities to develop and master health and life skills Skills to handle concurrent problems Integrating skills, good enough governance Internalized human rights experience
Dialogs and debates	*Judging appraisals* Static exchange aimed at sustaining status quo at separate moments in time Incidents elicit dialogs, requiring accounts for fiasco's	*Open, nurturing exchange* Inner dialogs and mutual exchange Metaphors, shared language Developing antennas, finetuning setpoints, increasing resilience and cultural sensitivity
Governance	*Risk and control management* Techno-economic governance, focused to symptom, risk, and cost containment Fragmented, siloed strategies. Alertness required to criminalization based on biased evidence	*Facilitating emergent growth in communities* Self-governance in triadic community networks. Dynamic exchange over time. Natural, mutual learning processes in relations. Sharing experiences, exploring social pathways toward thriving
Monitoring and research	*Limited information* “Evidence” based on selected populations or single recordings at a bad moment easily generates false beliefs on dangerousness or blameworthiness	*Whole population evidence* Meaningful real-life data in populations: dynamic mirrors and quality reviews foster local and (inter)national debates while keeping focus on fair space toward thriving

Acknowledging that contextualized narratives help to explore what matters, the seven transitions make current challenges for developing shared knowledge and mutual understanding accessible. The breakdown in separate transitions helps to delineate understandable, contextualized “packages” of relevant topics or tensions (87). Then, meaningful information helps to explore the ownership and power dynamics around conditions for growth. This eases transitions from action-oriented strategies based on malleability assumptions to process-oriented strategies based on emergent growth. The content is not specific for citizens with multiple interdependent needs. Instead, the gathering of information and the mutual attuning on possible opportunities apply to all citizens at all ecosystem levels.

### 6.3 Fostering mutual learning over social pathways

Social learning is considered important for working on culture change. As mentioned, shared experiences can foster shared values, social conscience, and social cohesion. In practice, social norms induce bias and sustain hierarchies. Fortunately, shared learning processes converge. Though popular in public health policy and research, in regular health-care services thinking in ecosystems is new. The pictograph shows how community networks can shape mutual learning processes in diverse socio-cultural settings.

**Figure 2:**
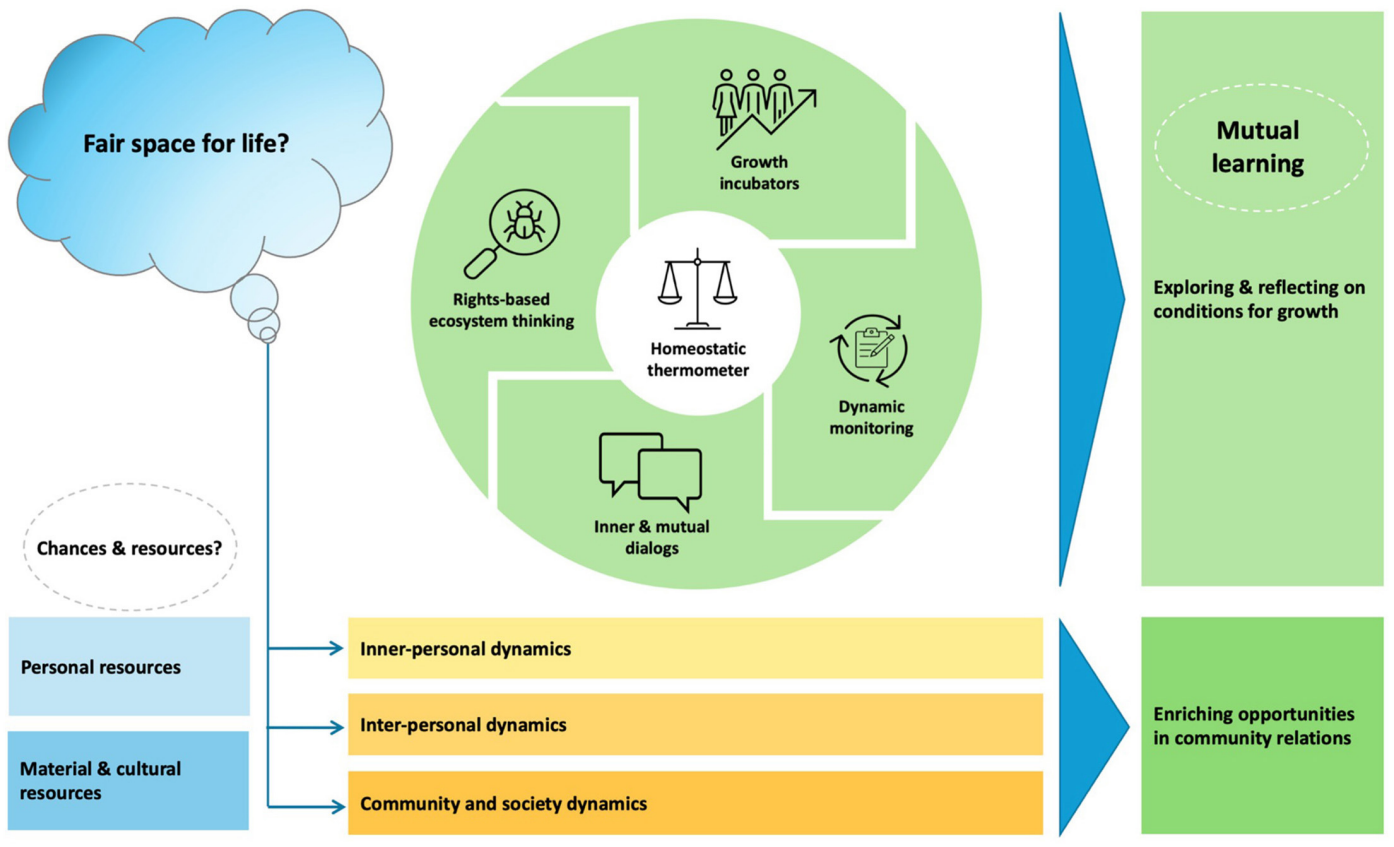
A dynamic rights-based ecosystem approach to enrich social pathways toward thriving.

Inherent in natural homeostatic mechanisms, communities monitor all kinds of safety threats. Figure 2 depicts how rights-based ecosystem strategies can improve resilience. The key question is how to promote “*fair space for life*.” Recognizing the human longing for health and happiness, the exploration starts with an appraisal of the actual resources and chances of health and well being. Like in Figure 1a, it examines physiological and safety needs, motives, and the place of citizens within communities. It explores how personal, material and cultural resources relate to actual contexts. How do present conditions affect the continuum between social decline and thriving?

The circle provides community networks with tools to facilitate mutual learning. It shows that the approach is suitable for integration into the Plan-Do-Study-Act cycles of the model for improvement. For planning goals, it is essential that community networks become familiar with this approach. *Rights-based ecosystem thinking* considers people together with their contexts. The logic examines how environmental conditions, health and needs patterns, and dynamics over social pathways interact.

In daily practices, families and other community relations serve as *growth incubators* to acquire and master fundamental health and life skills. All kinds of collaborative relations can foster shared learning and facilitate emergent growth. Exploring from various perspectives what happens opens community resources and alternative pathways.

For study aims, continual standardized monitoring tends to disregard relevant information in citizens groups with multiple interdependent needs. Instead, *dynamic monitoring* produces contextualized information on actual topics or tensions in community relations. It provides “food for thought” that makes growth dynamics or power dynamics transparent.

For action aims, such information stimulates reflection and shared learning. This elicits *inner and mutual dialogs* what happens and what matters. Simultaneously, it induces public dialogs in communities and regions. So, reflecting on collected data, the rights-based ecosystem filter fosters interactive co-creation dialogs what is essential for growth. It provides content and direction to future Plan-Do-Study-Act cycles in various settings.

Such dynamics affect and connect learning cycles in diverse settings. The lower section represents the interrelations at various ecosystem levels. Bonding, linking and bridging can foster mutual learning. This is necessary to deal with uncertainties and diversity. The dynamic approach over time assists networks in the framing and reframing what is important. This keeps the focus on fair space alive.

Mutual learning demands to readjust and finetune the *homeostatic thermometers* of growth incubators and other communities. The thermometers reflect the resources based on shared knowledge, values, memories, and reportoires in networks. They comprise an open, reflective, and collaborative attitude that is essential for fostering mutual learning in an interative approach.

The incubator concept prompts the lifelong character of mutual learning. Normalizing to explore and reflect on power dynamics in community relations enriches the opportunities for growth. Instilling such habits promotes empowerment, while fostering resilience of (citizens in) communities over time. Of course, this process of learning is not specific nor limited to citizens with multiple interdependent needs. The exchange between safe and public spaces eases disadvantaged groups to catch up. Simultaneously, it enhances the resilience of communities and the whole society.

Our study in the Netherlands demonstrates that it is impossible to simply fix multiple interdependent needs. Instead, the extent and circularity encourage community networks to incorporate dynamic, lifelong learning strategies to the continually changing obstacles and challenges for growth. Contact requires finetuned homeostatic antennas and a thorough understanding of essential conditions. Fortunately, community groups can provide safe spaces that facilitate reflection on what matters. Similarly, spontaneous encounters with (un)known others in public spaces also allow mutual learning on conditions for growth. The homeostatic thermometer represents the collective conscience in communities and recognizes the essence of patience and numerous chances that enable social pathways toward thriving.

## 7 Recommendations for practice, policy, and research

Inspired by the intention to improve community resilience, this study uses un unconventional approach. The systemic failure to match vital needs of the neediest is the most important finding. The rights-based ecosystem filter redefines the playing field and places the challenges in the middle of society. So, who is in charge and what is the next step?

Governments should secure essential conditions to enable growth and thriving for all. Practically, Dutch municipalities should coordinate the connections between various healthcare domains. Focusing on individual needs, the findings demand that we facilitate individual pathways. After all, the path toward thriving is a personal path. Embedded in personal living environments and lived experience, it interacts with opportunities provided by communities. Simultaneously, personal paths are embedded in community pathways. By nature, communities preserve the autonomy of its citizens and support disadvantaged fellows for whom thriving is not evident.

Acceptance of the never-ending interactions between people and their environments encourages to adopt dialog-driven, co-creative strategies for exploring what is reasonable and acceptable. Mutual re-explorations and re-adjustments are essential. This requires coherent, iterative, long-term improvement efforts in diverse systems and settings. The rights-based ecosystem filter provides communities and societies with process tools for expanding “fair space” and enrich opportunities toward thriving. This enables them to develop shared knowledge and actions.

For moving sustainably toward fair conditions for growth, our observations encourage us to avoid quick fixes. They stimulate to invest in process-based strategies for developing and fostering community resilience. They encourage us to enjoy reflection and mutual learning in daily life settings. Physical places are crucial to practice mutual learning and enrich our shared repertoires. Combining dynamic monitoring with the improvement model can help to keep the focus on fair space and the effectiveness of joint strategies alive.

This study reflects the interplay between research and practice. It subscribes the need of bottom-up strategies of mutual learning in diverse socio-cultural contexts. Additionally, it requires structural measures. The availability of affordable housing is not only a matter of good government, but a human right. Equally, it requires a rights-based policy to preserve fair space for all. Therefore, political choices and change-making strategies should not hinder, but promote mutual learning what enhances the natural autonomy and resilience of (citizens within) communities. Rights-based ecosystem monitoring can further modern understanding how psycho-socio-cultural factors over social pathways interact. We make five recommendations.

First, we recommend expanding the data-collection on thriving attempts of citizens in real-life settings. This study stimulates to focus on citizen groups with multiple interdependent needs, living at the edge of society and prone to downward spiraling. The Experience Sampling Methods collects data on the adaptational dynamics in daily life. It assesses user strengths and vulnerabilities in relation to environmental conditions. Experience Sampling data provides contextualized information on growth-enabling and impeding conditions over social pathways. Insights can facilitate mutual learning in communities and collaborative networks on meaning and socio-cultural conditions for growth.

Further, we recommend using transdiagnostic mental health assessments. These better describe the health patterns and make the circularity between symptoms and needs more transparent. The relation with long-term poverty, for example, nuances our understanding of marginalization and drop-out from more rewarding social pathways. By approaching disabilities as a social construct in a human rights context, this coutry-case advocates for an integrative strategy to improve adequate living standard. This framework can direct future improvement efforts in practice.

Inspired by prior long-term international studies, we recommend commissioning long-term Dutch cohort research for an updated modern understanding of the natural course of mental health recovery over time. Such cohort research can monitor changes in relation to personal, mutual, and collective learning processes that are relevant for resilience.

The profound impact of mental suffering in social interactions accentuates the need of fine-tuned antennas and safe spaces for bettering our understanding of conditions for growth. This requires investing in the education and empowerment of people with peer-lived experience. Collaborative learning requires an equal partnership of lived experience supplemented by process based coaching (from trained experts). Appointing people with lived experience as colleagues enriches mutual learning in practice, policy and research. User research in triadic community collaboratives will boost social learning strategies in diverse socio-cultural settings.

However, the collaborative challenges at various ecosystem levels substantiate the need for a profound culture change. Therefore, we recommend facilitating that community networks are educated at the use of the rights-based ecosystem filter to assess and implement improvements in pathways through care. Bottom-up user participation and collaboration in resource groups are essential. User-targeted investments would facilitate adequate servicing of user groups with multiple interdependent needs. Embedding dynamic care monitors in empowerment evaluations or community-based participatory research would expand our understanding of what works to shape environmental conditions for growth. It facilitates mutual learning, for instance how safe and public spaces affect growth. Besides, insight on the interplay between safe and public spaces can learn what helps to enrich pathways toward thriving for all.

## Data Availability

The original contributions presented in the study are included in the article/Supplementary material, further inquiries can be directed to the corresponding author.

## Footnotes

^1^EPA is the Dutch acronym for Severe Mental Illness (SMI).

^2^In the “vital needs” paper, Physical Monitoring Needs reflect regular monitoring needs because of chronic physical conditions, including the consequences of sex work and hard drug use. This explains the higher prevalence, compared to the “health patterns” paper.

^3^Fair Space (36), table 4.

^4^Vital Needs (34), table 4.

^5^Fair Space (36), appendix B.

^6^Source: https://www.britannica.com/topic/epistemology.
