# Supplementary material for: Fair space? Community relations as a booster for improving resilience
Source: Front Public Health. 2025 Jul 8;13:1585985. doi: 10.3389/fpubh.2025.1585985 (PMC12279791; doi:10.3389/fpubh.2025.1585985)
Supplement: Supplementary file 1 [file Table_1.docx]

Supplementary table related to **Fair Space? Community relations as a booster for improving resilience**

doi: 10.3389/fpubh.2025.1585985

| **TABLE 1 Topic, research focus, and knowledge output** | | | | |
| --- | --- | --- | --- | --- |
| ***Topic*** | ***CP*** | ***Research focus*** | ***Knowledge output*** | ***Research aim*** |
| ***A rights-based ecosystem approach*** | 2.1 to 2.3 | Local health reviews aimed to support  care planning in various settings | Participatory research in a rights-based ecosystem approach |  |
|  |  | Dual snowball sampling  (settings & individuals within settings) | Representative population sample |  |
|  |  | Comprehensive, recovery-oriented vulnerability-strength assessments in real-life settings | Structured, systematical health & needs assessments of individual service users  covering all life domains |  |
| ***Care of***  ***homeless citizens in a modern welfare state*** | 3.1 | Generic, transdiagnostic approach  of health and needs | Health patterns: Burden, overlap, and interrelations  (physical, mental, addiction, intellectual issues) | Describe the health of service users, including different health aspects and comorbidity |
|  |  | Mental health-related needs assessment based on the Dutch consensus SMI/EPA | Real daily-life impact: distinguishing conditional from intensive needs |  |
|  | 3.2 | Vital needs in Maslow’s hierarchy  on top of the need of a home | Needs patterns: Burden & overlap of concurrent vital needs (physical, mental, administration, paid work) | Analyze patterns of marginalization & disclose care gaps in interactions of users and systems |
|  |  | Actual care from local service networks  in relation to present needs | Fairness appraisal:  Adequate care conform to professional standards? |  |
| ***Mutual learning what matters for improving resilience*** | 3.3 | Longitudinal follow up of interactions around 15 cases in a local experiment for ‘deadlocked cases’:  What works to establish working relations?  What goes wrong between users and systems? How can we create stable conditions for growth? | In-depth exploration:  Dynamics & tensions in interactions  Obstacles & opportunities in community relations  Essentials for successful collaboration  Demonstration of mutual learning | Identify conditions to promote pathways toward thriving in marginalized populations with interdependent needs |
|  | 3.4 | Impact of continuing distress on relations  (self, neighbors, formal caregivers, community/society) | Distress-induced dynamics in relations  Conditions for growth & citizenship over time  Keys & opportunities of a dynamic care monitor |  |

Abbreviations CP = Corresponding paragraph
