# Supplementary material for: Fair space? Community relations as a booster for improving resilience
Source: Front Public Health. 2025 Jul 8;13:1585985. doi: 10.3389/fpubh.2025.1585985 (PMC12279791; doi:10.3389/fpubh.2025.1585985)
Supplement: Supplementary file 2 [file Data_Sheet_1.docx]

Supplementary material related to
**Fair Space? Community relations as a booster for improving resilience**

doi: 10.3389/fpubh.2025.1585985

| ***Amin*** *Through his sad, depressed look, Amin (43) appeared a friendly, social man. In fluid Dutch, he shared his history: “Twenty years ago, I was working in the tourist industry of my African home country. My marriage marked my start here. I had a paid job as a social worker, until flashbacks started to disturb my life… About ten years ago, I lost my job. I received antipsychotics for a year. After a relation breakdown, I became homeless...” He explains that he was living in a protected living facility for several years, when he got involved in money mule practices: “At that time, I didn’t have much money. For a small rent, I let my car to someone of my neighborhood. When the man failed to return it, I notified the police... Later, they found my car back on a scrapheap in another city. It was still on my name. My debts towered, through the unpaid fines and tax assessments. So, two years ago, I fell into homelessness again.” A silent tear runs over his cheek. With a deep breath, he continues: “I wish that that ‘car business’ would be finished... I really hope that this unfairness will end. I want my life back… For long, I had a respected job. Later, I was volunteering in this neighborhood. Nowadays, I actively withdraw myself from social networks. Driven by social contact needs, I go to the city center to play the drums. But if people recognize me, I’m ashamed... I’m desperate... They told me that I’m on the waiting lists for a living facility. After one year, I even didn’t have an intake yet.”*  ***Amanda*** *She (43) grew up in a big, warm-hearted family. After finishing elementary school, she became responsible of the family household. Her parents and older sister were working outdoors. At her eighteenth, she left South America to live with her Dutch boyfriend. Ten years later, a breakdown with domestic violence resulted in homelessness. Her smile breathes her positive attitude, while recounting the meaning of her friends: “They helped me building a new life! They assisted me to find a new job and a home.” Using her housekeeper skills, she accepted jobs in hotels. She used pragmatic strategies for compensating functional illiteracy. She regularly switched between apartments and rooms to overcome jobless episodes. Three times, she fell into homelessness: “I heard voices and had horrible dreams… Now, my psychotic complaints are under control. I’m looking for a new job and a new home. I’m volunteering in the kitchen. I can live on my own, but I’ll accept a protected living place, too. I want to leave the shelter as soon as possible!”* |
| --- |

| ***Fred*** *Fred (55) is an intelligent man with strong social values. After a successful international career, he started his own company as a real estate agent. He became homeless during the Dutch mortgage crisis: “I was enforced to make use of the shelters. Because of my huge debts, I have no chances in the rental market. In fact, I don’t need any support. A rented home would suffice… Fortunately, the municipal trajectory gives perspective on social housing…” In the shelters, he daily assists his fellows: “I compensate for the pain of my own social defeat. Now I help others to fight for their social rights. It distracts me to address inequalities and unfairness, while advocating for social justice….”  During the local health review, Fred reached out to me: “As soon as I have an address of my own, I will establish a foundation for advocacy in the field of social rights. When is the review report made public?”* |
| --- |

| ***Paul*** *Tensed at the start, he (50) recalls his life quietly: “Like my father, I have a bipolar disorder. Living happily with my wife and children, I started throwing down chairs from the balcony. I was forty, when the first admission took place… Consequently, I lost my partner, my family, and my home…. To manage difficulties in keeping a regular job, I started working as a craftsman with my friend and moved to a caravan. Then, my camping neighbors began to exploit criminal activities. I started drinking, to stand the threatening atmosphere. Finally, I was totally in panic. I had to flight. I became homeless when my caravan burnt down, somewhere along the road…  My friend stays with me through thick and thin. He has confidence in me. He invited me to start working again. But I first need to overcome what happened. I need an income and a place on my own… Instead, I’m forced to accept a place in a protected living, where I’m involuntarily exposed to my housemates’ substance using behavior…” He ends confident: “I need to stand it. I have the prospect of an apartment in another city over some time! I have a good relation with my psychiatrist. I can manage things by myself. I don’t need any help of social services at all.”*  ***Paula***  *The tall Paula (26) engages openly in contact. Light-heartedly, she starts to share: “My mom was born in South America. After the divorce, her new partners didn’t treat me as their child. I was sent to special education, as I was a ‘difficult’ child. Still, I got my school certificate and had several jobs. Yet, when I was eighteen, my mom threw me out. Hospital admissions alternated with stays at her home... I don’t know exactly how long, but I guess that I’m in the shelter for two or three years now.” Then, with a mocking smile: “I’m fond of comics and bad guys! I’m a bit obstinate. I enjoy all kinds of thrills, so I’m quite impulsive. I easily trust people, so I’m easily abused… Thus, my mom prefers to keep distance. Likewise, I have no contact at all with family or prior friends.” More seriously: “I drink a lot. Daily, I’m using strong drinks. Daily, I’m using speed. Besides, I need cannabis to find my rest…” Firmly, she confesses: “I want to live my life instead of being lived. I’m longing for a regular life… Thus, I’m motivated for meds and therapy, as proposed by the community treatment team. With Individual Placement Support, I obtained a job at a call center. I’m on the waiting list for a dual-diagnose team with Housing First!”* |
| --- |

| ***Chris***  *Chris (19) engages openly. Checking the lock of the cupboard, he apologizes: “I’m easily distracted. There’s continuous turmoil in my head… Since I was four, my substance abusing father ‘corrected’ my restlessness with his fists and all sorts of names. I started beating classmates. After kicking a teacher, I was removed from school. I didn’t match with the new partner of my mom, so I ran away. The intensive treatment program was better, but the barred windows of the institution could not prevent experimenting with drugs. Since I was twelve, I’ve used all kinds of drugs, you know.” Chris was houseless since he was ten. Recently, he landed on the streets after an aggressive incident. He reported precisely on his situation, needs, and the risks due to his behavior: “The directive tone of the facility worker triggered me. On the weekends, I visit my mom, 200 kilometers from here. I trust her, but not completely. The rest of the time, I’m hanging around… The last fortnight, I met over 400 people, you know. I’m still alone. The municipal intake is running****,*** *and I’m on various waiting lists for court ordered programs. I’m looking forward to going to school again. How many months the procedures will take? My substance use is extreme. I’m acting on impulse very easily. Yesterday, we were on top of a high rise….”*  ***Simon***  *Since his youth, Simon’s life was unstable. After a clash with his parents, he became homeless. Over a decade, long (in)voluntary admissions alternated with homelessness. In the perspective of mental health services, well-intended treatment plans were obstructed by his behavior, his refusal to use antipsychotics and his unwillingness to engage. Simon explained his aggression as a response of despair and frustration to enforced treatment: “Clinics make people go crazy. I don’t want to be patronized; I want to be taken seriously.” Considered a ‘deadlocked case’, his reputation limited fulfilment of care needs. Instead, the police filed dozens of aggressive incidents. Putative danger and supposed difficulties excused mental health services for discontinuing care. The S-team was added in a desperate attempt to enable recovery. From the start, the S-team combined normal, interhuman behavior with pragmatic support in daily life issues. Soon, he had a place to live on his own. This enabled him to achieve fundamental health and life skills. Still, visits of former companions incite him to use drugs. Despite much coordination, his life remained unstable. Parallel care plans from separate sectors proved insufficient to break the downward spiraling of psychotic fear, presumed dangerousness, restraints, and recurrent hospitalizations.  The start with depot antipsychotics, and the mentoring of a relative marked the onset of empowering community relations with his neighbors, caregivers and other parties. Complaining about prior nuisance of the police squad, the neighbors explained: “Simon is a decent boy. The nuisance was caused by his friends. We would have averted the risk of the next admission if we had been able to reach him!” Since then, no additional admissions or police records were filed.* |
| --- |

‘Amin’, ‘Amanda’, ‘Paul’, ‘Paula’, ‘Chris’, and ‘Simon’ are imaginary names.
Fred is the real name of a study participant. He gave explicit permission for using his name.
